# Cancer and Takotsubo syndrome: from rarity to clinical practice

**DOI:** 10.1002/ehf2.13741

**Published:** 2021-12-06

**Authors:** Kalliopi Keramida, Dimitrios Farmakis, Gerasimos Filippatos

**Affiliations:** ^1^ Cardiology Department, General Anti‐Cancer Oncological Hospital Agios Savvas Athens Greece; ^2^ University of Cyprus Medical School Nicosia Cyprus; ^3^ Department of Cardiology National and Kapodistrian University of Athens School of Medicine, University Hospital Attikon Athens Greece

Takotsubo syndrome (TTS) or stress cardiomyopathy in cancer patients has been mainly reported either as a cardiotoxic effect of antineoplastic treatment or a complication of specific tumors, such as pheochromocytoma and paraganglioma.^1^ The pathophysiology of TTS in patients with and without cancer is rather complicated and vague. Although TTS is underdiagnosed in oncological patients, cancer and TTS co‐exist more often than previously believed. There is evidence that oncological patients have higher incidence of TTS compared to the general population, with a mean incidence around 53 in 100.000 chemotherapy‐related hospitalizations versus 20.4 in the general population, whereas its incidence in patients hospitalized with suspicion of acute coronary syndrome is around 2%.^2^, ^3^, ^4^ Conversely, several studies and registries show that the prevalence of neoplasms (occult or overt, antecedent or active) is increased in patients with TTS compared to individuals with the same age and sex without the syndrome with a range of 4–29%, both at the time of diagnosis and during follow‐up.^5^, ^6^ It is noteworthy that TTS incidence in cancer patients is similar in men and women, while in the general population, there is a well‐established predilection for women.^2^, ^4^

The triggers of TTS in cancer are diverse and multiple (*Figure* *1*). The neoplasm itself, anticancer therapies including chemotherapy, targeted therapies, combination therapies, radiotherapy, radioablation therapy, surgery, diagnostic and therapeutic procedures, genetic predisposition, emotional and psychosocial stress, acute illness like infection or acute respiratory failure, and cancer pain are the most explored ones.^1^, ^2^, ^7^, ^8^, ^9^, ^10^, ^11^ These causative and predisposing factors justify the ‘multi‐hit hypothesis’ not only for heart failure, but also for TTS in cancer patients.^12^ There are several proposed pathophysiological mechanisms responsible for TTS, with activation of sympathetic nervous system and coronary vasospasm being the most popular (*Figure* *1*).

**Figure 1:**
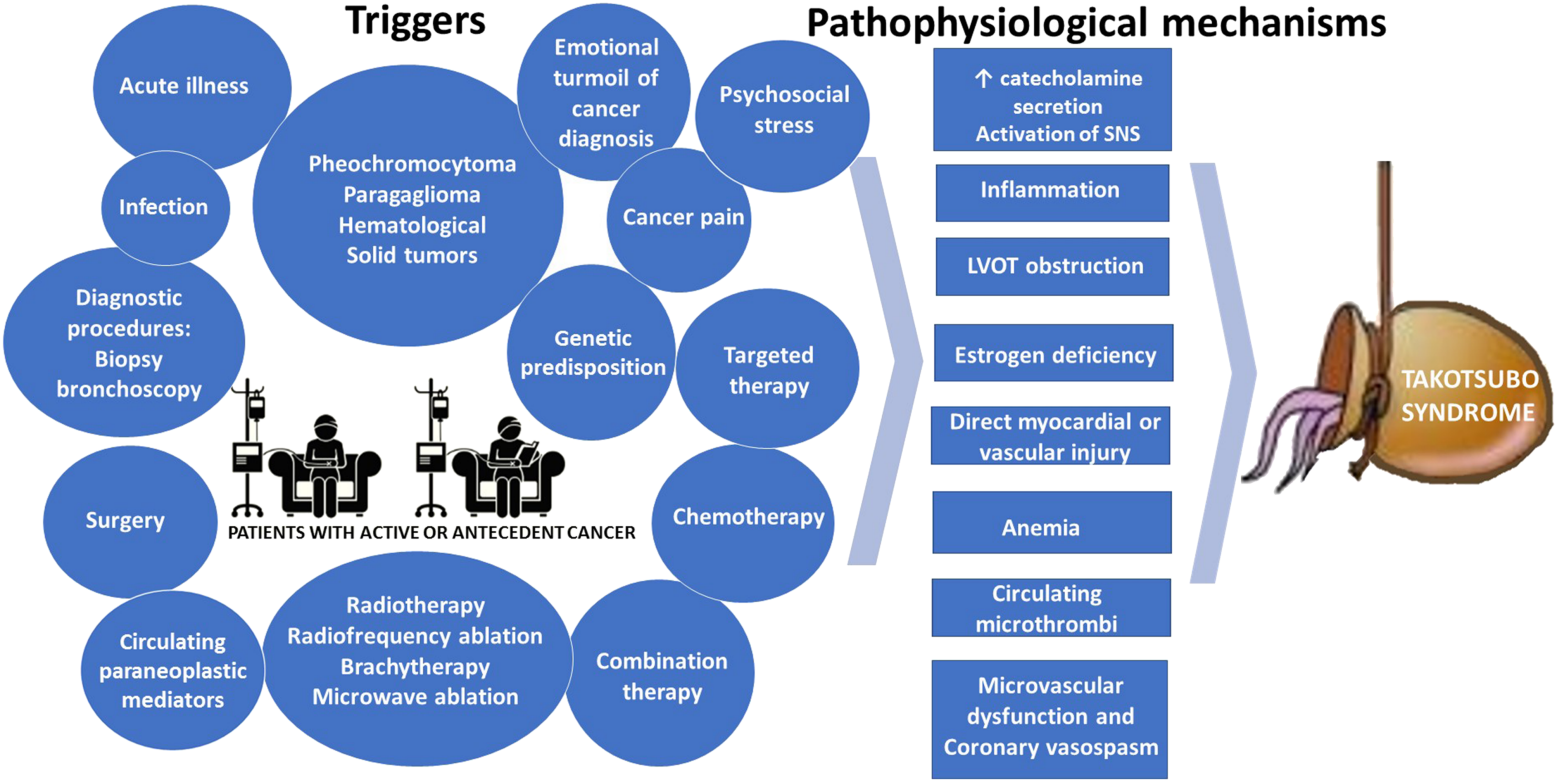
Potential triggers and proposed pathophysiological mechanisms of Takotsubo syndrome in oncological patients.

Several studies show that patients with solid tumours are more likely to develop TTS compared with those with haematologic malignancies.^13^ The InterTAK Registry reported that the most common malignancy associated with TTS is breast cancer, followed by tumors of gastrointestinal system and then respiratory track, and so forth.^6^ Javaid and colleagues^14^ report the results of a large cohort study, based on a US national inpatient sample, having included 4.7 million patients hospitalized with active cancer.^14^ The authors found a significant prevalence of TTS in these patients of 12%, while they further investigated the association between primary tumor type and the risk for TTS using propensity‐score adjustment and machine learning. According to their findings, only breast and lung cancer were associated with a significantly increased likelihood of TTS. In addition to tumor type, the stage of cancer is also an important factor to be considered, as TTS seems to be more prevalent among patients with advanced or recurrent disease.^13^

Despite the fact that anticancer treatment is considered the main culprit for TTS development, the effect of antineoplastic treatment was not taken into account by Javaid and colleagues in their propensity‐matched, machine‐learning analysis that focused on the primary malignancy type.^14^ Several case reports and registries have implicated a long list of systemic anticancer therapies, including chemotherapy and targeted therapies (*Table* *1*). Among them, 5‐fluorouracil capecitabine, anthracyclines, trastuzumab, and immune checkpoint inhibitors, either alone or in combination with and without concurrent radiotherapy, have been mostly associated with TTS.^2^, ^15^, ^16^ Among cancer patients exposed to these therapies, those with additional risk factors are more prone to develop TTS. Javaid *et al*. and previous investigators have identified a series of risk factors including female sex, age older than 45 years, and cardiovascular and non‐cardiovascular comorbidities, such as hypertension, dyslipidaemia, anaemia, lung and neurologic disease.^6^, ^14^, ^15^, ^17^

**Table 1:** Antineoplastic agents that induce Takotsubo syndrome

Antineoplastic agents	Indicative references
**Antimetabolites**	
5‐Fluorouracil	Lim SH, *et al*. Case Rep Oncol Med. 2013;2013:206765. Valero M, *et al*. J Geriatr Oncol. 2020 Nov;11(8):1337–1339. Kobayashi N, *et al*. J Nippon Med Sch. 2009 Feb;76(1):27–33. Ozturk MA, *et al*. Blood Coagul Fibrinolysis. 2013 Jan;24(1):90–4. Kumar D, *et al*. Cureus. 2021 Mar 22;13(3):e14049. Basselin C, *et al*. Pharmacotherapy. 2011 Feb;31(2):226. Knott K, *et al*. Int J Cardiol. 2014 Dec 15;177(2):e65–7. De Pasquale MD, *et al*. BMC Pediatr. 2016 Jul 19;16:99. Stewart T, *et al*. Intern Med J. 2010 Apr;40(4):303–7. Gianni M, *et al*. Blood Coagul Fibrinolysis. 2009 Jun;20(4):306–8. Coen M, *et al*. BMC Cancer. 2017 Jun 2;17(1):394. Kobayashi N, *et al*. J Nippon Med Sch. 2009;76(1):27–33. Cheriparambil KM, *et al*. Angiology. 2000;51(10):873–8. Dalzell JR, *et al*. Anti‐Cancer Drugs. 2009;20(1):79–80. Grunwald MR, *et al*. J Clin Oncol. 2012, 10;30(2):e11–4.
Capecitabine	Bhardwaj PV, *et al*. Perm J. 2019;23:18–245. Qasem A, *et al*. Am J Ther. 2016 Sep–Oct;23(5):e1188–92 Abdelmaseih R, *et al*. Curr Probl Cardiol. 2021 Aug;46(8):100854. Powers A *et al*. J Am Coll Cardiol 2020;75(11 Supplement 1):3279. Sabharwal S, *et al*. Chest 2018;54(4 suppl):107A. Sakamoto T, *et al*. J Cardiac Fail 2016;Vol. 22. No. 9S September. Stewart T, *et al*. Intern Med J 2010;40(4):303–7. Y‐Hassan S, *et al*. Cardiovasc Revasc Med 2013;14(1):57–61. Ang C, *et al*. Curr Oncol 2010;17(1):59–63. Klag T, *et al*. Clin Res Cardiol. 2014 Mar;103(3):247–50. Endo A, *et al*. Int Heart J. 2013;54(6):417–20. Molteni LP, *et al*. Breast J. 2010;16(s1):S45–8.
Cytarabine	Baumann S, *et al*. Oncol res Treat. 2014;37(9):487–90. Madias JE. Oncol Res Treat. 2015;38(3):125.
**Anthracyclines/topoisomerase II inhibitors**	
Doxorubicin	Mubarak G, *et al*. Clin Case Rep. 2019 Nov 8;7(12):2466–71.
**Plant alkaloids/topoisomerase I inhibitors**	
Irinotecan	Kim L, *et al*. J Invasive Cardiol. 2008 Dec;20(12):E338–40.
**HER2 inhibitors**	
Trastuzumab	Burgy M, *et al*. Anticancer Res. 2014;34(7):3579–82. Khanji M, *et al*. Clin Oncol (R Coll Radiol). 2013;25(5):329. Matsumoto T, *et al*. Int Cancer Conf J. 2019 Dec 6;9(1):28–31. Lees C, *et al*. Oncologist. 2019 Feb;24(2):e80–2.
**Platinum compounds**	
Cisplatin	Hannan A, *et al*. Pak J Med Sci. 2018 Jul–Aug;34(4):1030–3.
**Other monoclonal antibodies**	
Rituximab	Smith SA, *et al*. Heart Fail Clin. 2013;9(2):233–42. X Ng KH, *et al*. BMJ Case Rep. 2015;2:2015.
Cetuximab	Kim L, *et al*. J Invasive Cardiol. 2008 Dec;20(12):E338–40.
**Anti‐VEGF antibodies**	
Bevacizumab	Franco TH, *et al*. Ther Clin Risk Manag. 2008;4(6):1367–70.
**Immune checkpoint inhibitors (ICIs)**	
Ipilimumab	Geisler BP, *et al*. J Immunother Cancer. 2015;3:4.
Pembrolizumab	Ederhy S *et al*. Eur J Heart Fail. 2019 Jul;21(7):945–7.
Nivolumab	Ederhy S *et al*. Eur J Heart Fail. 2019 Jul;21(7):945–7.
Ipilimumab + nivolumab	Oldfield, *et al*. BMJ Case Reports vol. 14,2 e237217. 1 Feb. 2021.
**Multitargeted tyrosine kinase inhibitors (TKIs)**	
Ibrutinib	Giza DE, *et al*. Eur Heart J Case Rep. 2017 Nov 2;1(2):ytx006.
**VEGF signalling pathway inhibitors**	
Axitinib	Ovadia D, *et al*. J Clin Oncol. 2015, 1; 33(1):e1–3.
Sunitinib	Numico G, *et al*. J Clin Oncol. 2012, 20;30(24):e218–20
Regorafenib	Arunachalam K, *et al*. R I Med J (2013). 2020 Dec 1;103(10):40–3.
**Miscellaneous agents**	
Combretastatin	Bhakta S, *et al*. Clin Cardiol. 2009;32(12):E80–4.
**Combination therapy**	
Cisplatin + docetaxel	Toyooka S, *et al*. Gen Thorac Cardiovasc Surg. 2012 Sep;60(9):599–602.
Cytarabine + daunorubicin	Goel S, *et al*. World J Clin Cases. 2014 Oct 6;2(10):565–8.
Vinorelbine + capecitabine	
FOLFOX (folinic acid, fluorouracil, and oxaliplatin)	Ali S, *et al*. Journal of Hospital and Medical Management 2021. Sundaravel S, *et al*. Case Rep Cardiol. 2017;2017:8507096. Basselin C, *et al*. Pharmacotherapy. 2011;31(2):226. Yeh ET, *et al*. Circulation. 2004;109(25):3122–31. Stewart T, *et al*. Intern Med J. 2010;40(4):303–7. Parker WB, *et al*. Pharmacol Ther. 1990;48(3):381–95. Ozturk MA, *et al*. Blood Coagul Fibrinolysis. 2013;24(1):90–4.
mFOLFOX6	Saif MW *et al*. Cureus. 2016 Sep 14;8(9):e783.
Gemcitabine + vinorelbine	Özbay B, *et al*. Turk Kardiyol Dern Ars. 2021 Jan;49(1):72–5
Carboplatin + pemetrexed + pembrolizumab	Khan NAJ, *et al*. Cureus. 2020 Jul 27;12(7):e9429.
Paclitaxel + hydroxyurea + 5‐FU and radiotherapy	Schweizer MT, *et al*. J Clin Oncol. 2011, 10;29(20):e598–e600.
Cisplatin + 5‐FU + methotrexate + mitomycin + bleomycin	Coen M, *et al*. BMC Cancer. 2017 Jun 2;17(1):394.
Combretastatin + cisplatin + doxorubicin	Cheungpasitporn W, *et al*. J Renal Inj Prev. 2017;6(1):18–25.
Rituximab + methylprednisolone	Ovadia D, *et al*. J Clin Oncol. 2014;33(1):e1–3.
Trastuzumab + docetaxel + carboplatin	Norwood TG, *et al*. J Immunother Cancer. 2017;5(1):91.
Cisplatin + 5‐FU	Dalzell JR, *et al*. Anti‐Cancer Drugs. 2009;20(1):79–80. Tsibiribi P, *et al*. Bull Cancer. 2006;93(3):10027–30.
DCF + FOLFIRI	Becker K, *et al*. Drugs. 1999;57(4):475–84.
5‐FU + levofolinate	Naranjo CA, *et al*. Clin Pharmacol Ther. 1981;30(2):239–45.
Daunorubicin + cytarabine + doxorubicin	Voit, J., *et al*. BMJ Case Reports, 2018, bcr2018226378.
Pembrolizumab + trastuzumab	Anderson RD, *et al*. Int J Cardiol. 2016 Nov 1;222:760–1
Ipilimumab + nivolumab	Ederhy S, *et al*. JACC Cardiovasc Imaging. 2018 Aug;11(8):1187–90.
Trastuzumab + pertuzumab + nab‐paclitaxel	Lees C, *et al*. Oncologist. 2019 Feb;24(2):e80–2.

The median time of TTS onset is 2 days (1–150) after the beginning of therapy.^15^ The typical clinical presentation of TTS is that of chest pain or dyspnea during or after treatment, but in 26.8% of patients, cardiogenic shock may be the first manifestation being potentially lethal.^15^, ^18^ Other complications include respiratory failure, pulmonary edema, arrhythmias, and cardiac thrombus or even cardiac arrest.^13^ Although TTS is usually a benign, self‐restricted syndrome, its prognostic effect in oncological patients is questionable. It can lead to interruption of anticancer therapy, which may adversely affect oncological outcome. Subsequently, rechallenging with the culprit antineoplastic therapy constitutes a major clinical dilemma with limited data on safety. Furthermore, previous reports have shown that the co‐existence of cancer and TTS results in increased length of stay and all‐cause, in‐hospital, and long‐term mortality.^11^, ^19^ Consequently, experts suggest to perform a risk stratification after the diagnosis of TTS^20^ in order to identify high‐risk patients for complications, which deserve aggressive treatment and extended surveillance. Interestingly, Javaid and colleagues found that TTS was associated with reduced mortality only in combination with breast cancer and not other tumor types.^14^

The diagnosis of TTS may be missed in cancer patients given the increasing frequency and wide range of cardiotoxic effects of systemic antineoplastic treatments, such as left ventricular dysfunction, heart failure, myocarditis, and acute myocardial infarction. High suspicion is essential for early diagnosis and improved outcomes with lower mortality rates and healthcare costs. A multifactorial risk model would enhance the inclusion of TTS in the differential diagnosis of treating physicians in oncological patients. Diagnosis can be very challenging in end‐stage patients on palliative care with subsequent therapeutic dilemmas. Analysis of big data from large oncological cohorts or registries, using machine learning and other sophisticated approaches, as in the case of the current study by Javaid and colleagues, can identify those cancer patients with the highest pre‐test probability to have TTS in order to facilitate timely diagnosis and efficient treatment. On the other hand, translational studies will be valuable in order to understand the underlying pathophysiological mechanisms that lead to TTS, revealing specific targets for prevention and treatment, and define whether rechallenge with the same or similar anticancer agents is safe and possible.

## Conflict of interest

The authors declare that they have no conflict of interest related to the submitted manuscript entitled ‘Cancer and Takotsubo syndrome: from rarity to clinical practice’.
